# Supplementary figures and images for: The effectiveness of early start of Grade III response to dengue in Guangzhou, China: A population-based interrupted time-series study
Source: PLoS Negl Trop Dis. 2020 Aug 7;14(8):e0008541. doi: 10.1371/journal.pntd.0008541 (PMC7444500; doi:10.1371/journal.pntd.0008541)

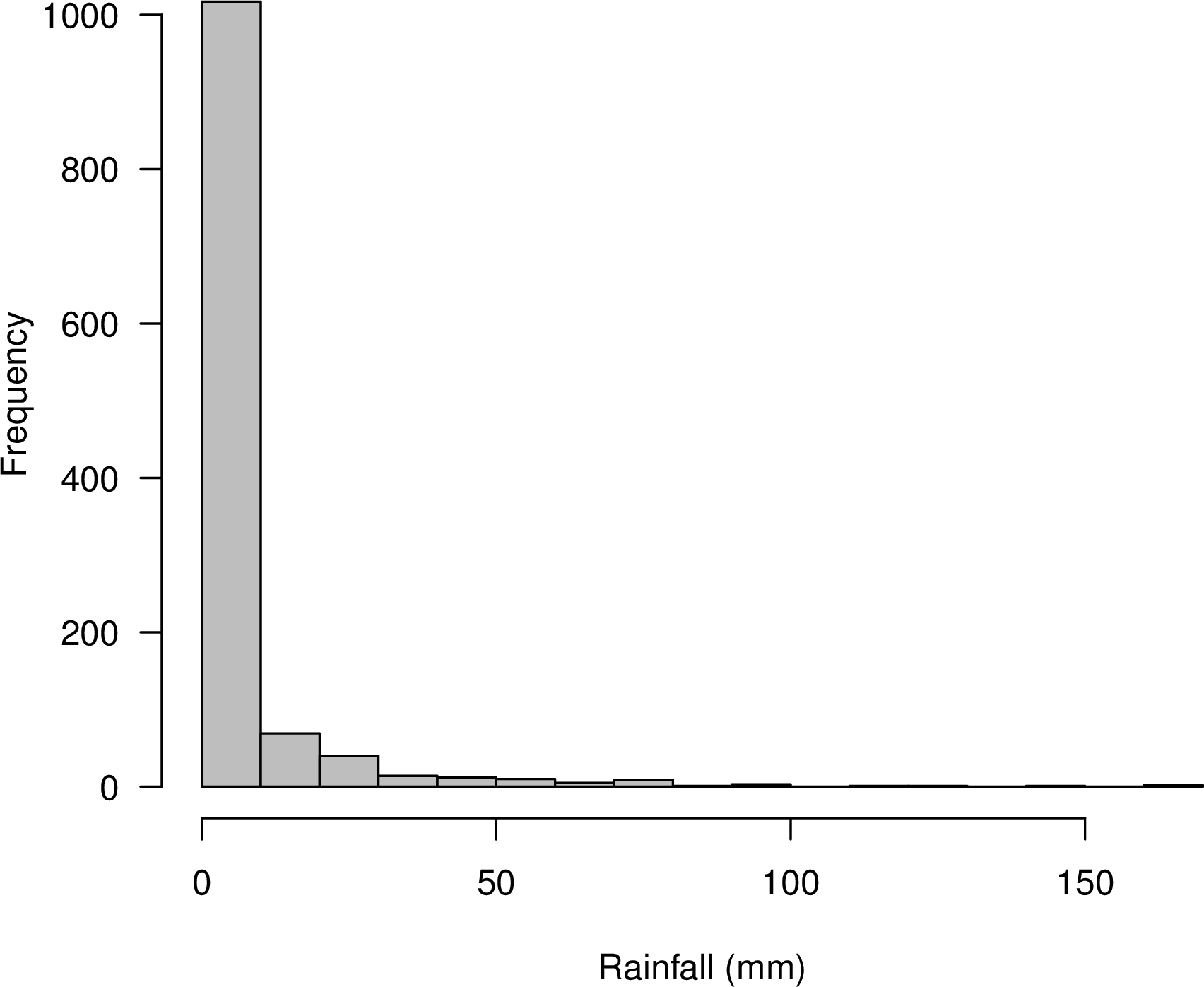

Supplement: S1 Fig — (TIF) [file pntd.0008541.s001.tif]

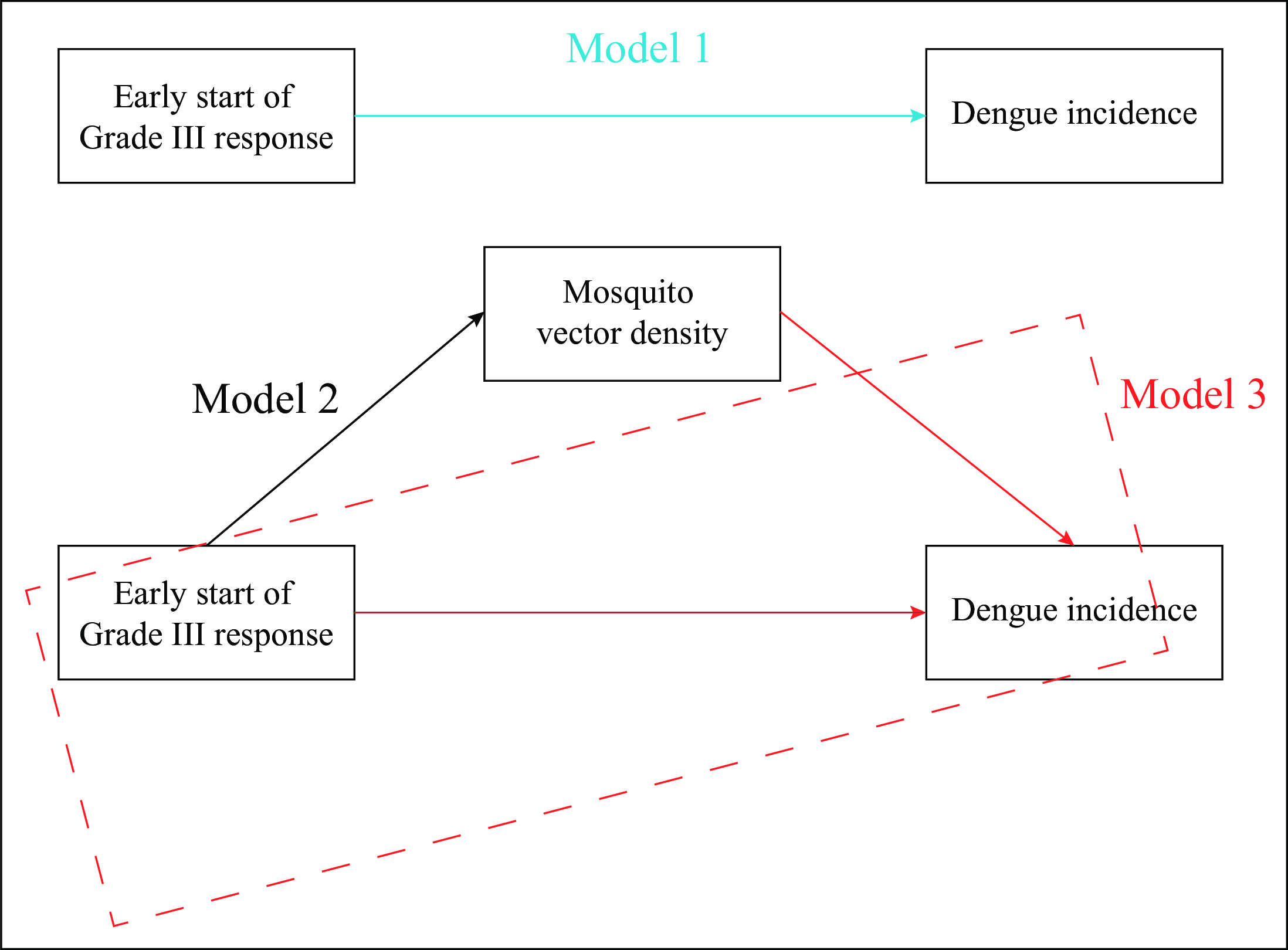

Supplement: S2 Fig — (TIF) [file pntd.0008541.s002.tif]

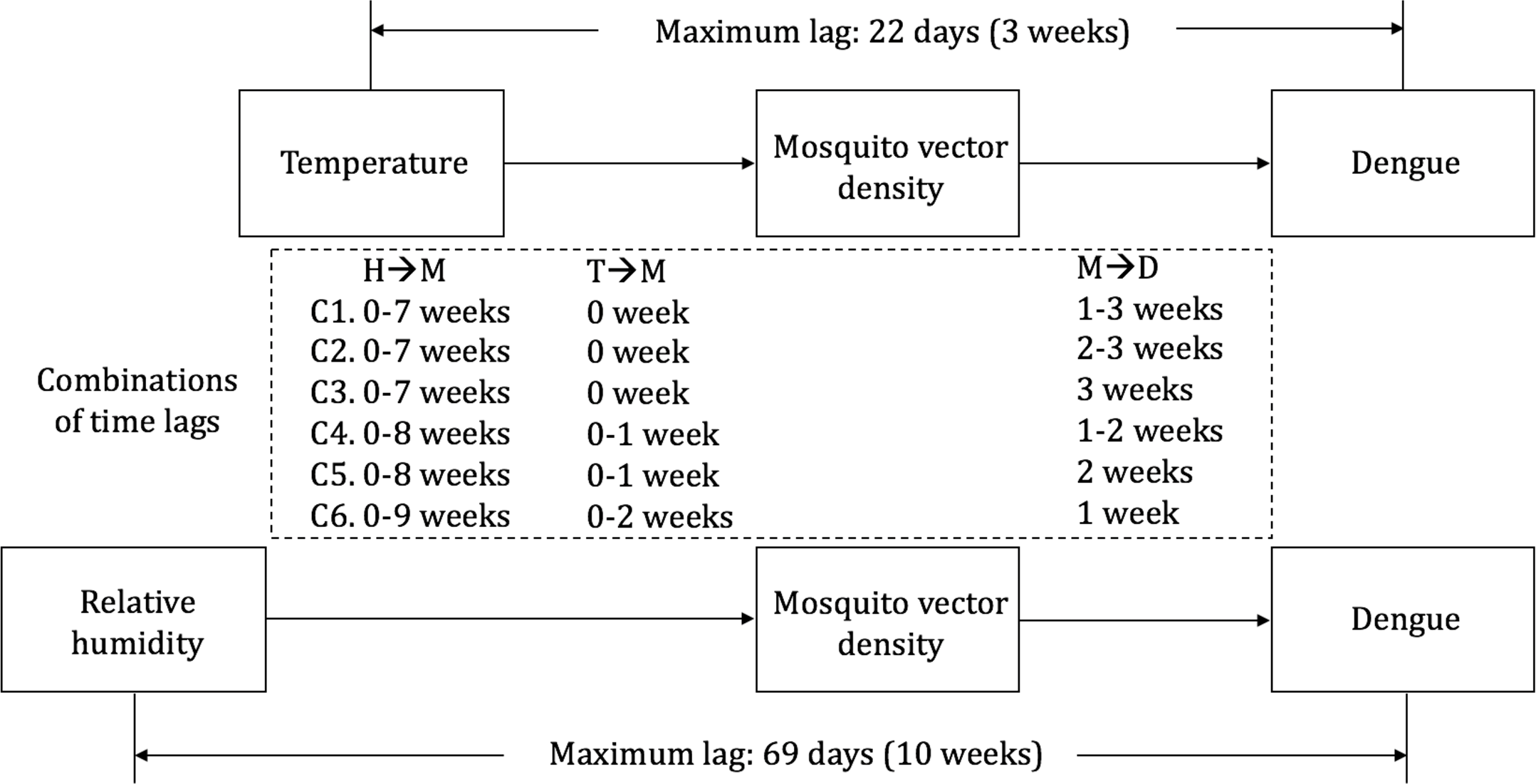

Supplement: S3 Fig — Six combinations of time lags (C1-C6) were shown in the dashed line rectangle. H→M, T→M, and M→D represent the time lags between relative humidity and mosquito vector density, between temperature and mosquito vector density, between mosquito vector density and dengue incidence, respectively. (TIF) [file pntd.0008541.s003.tif]

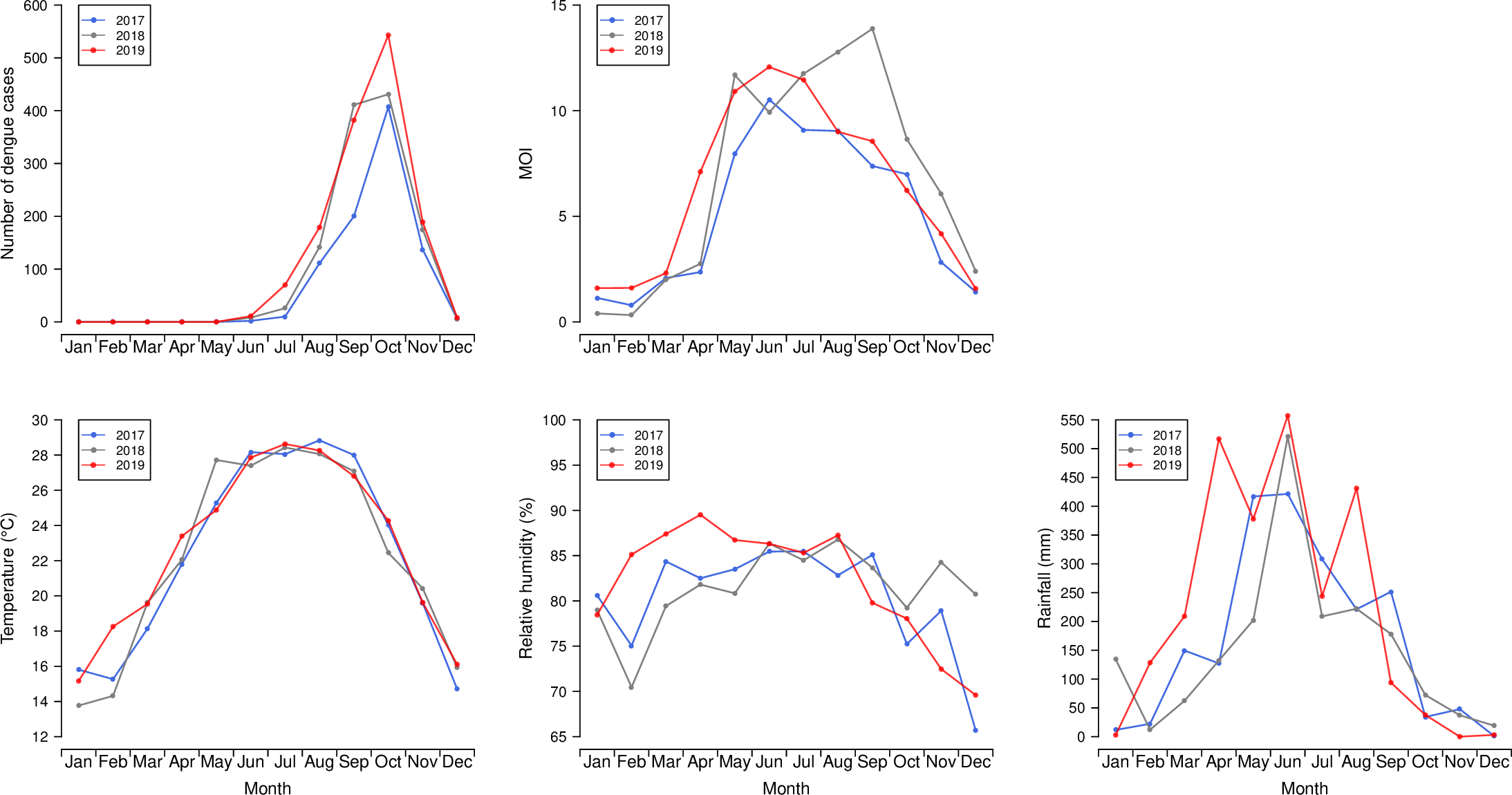

Supplement: S4 Fig — (TIF) [file pntd.0008541.s004.tif]

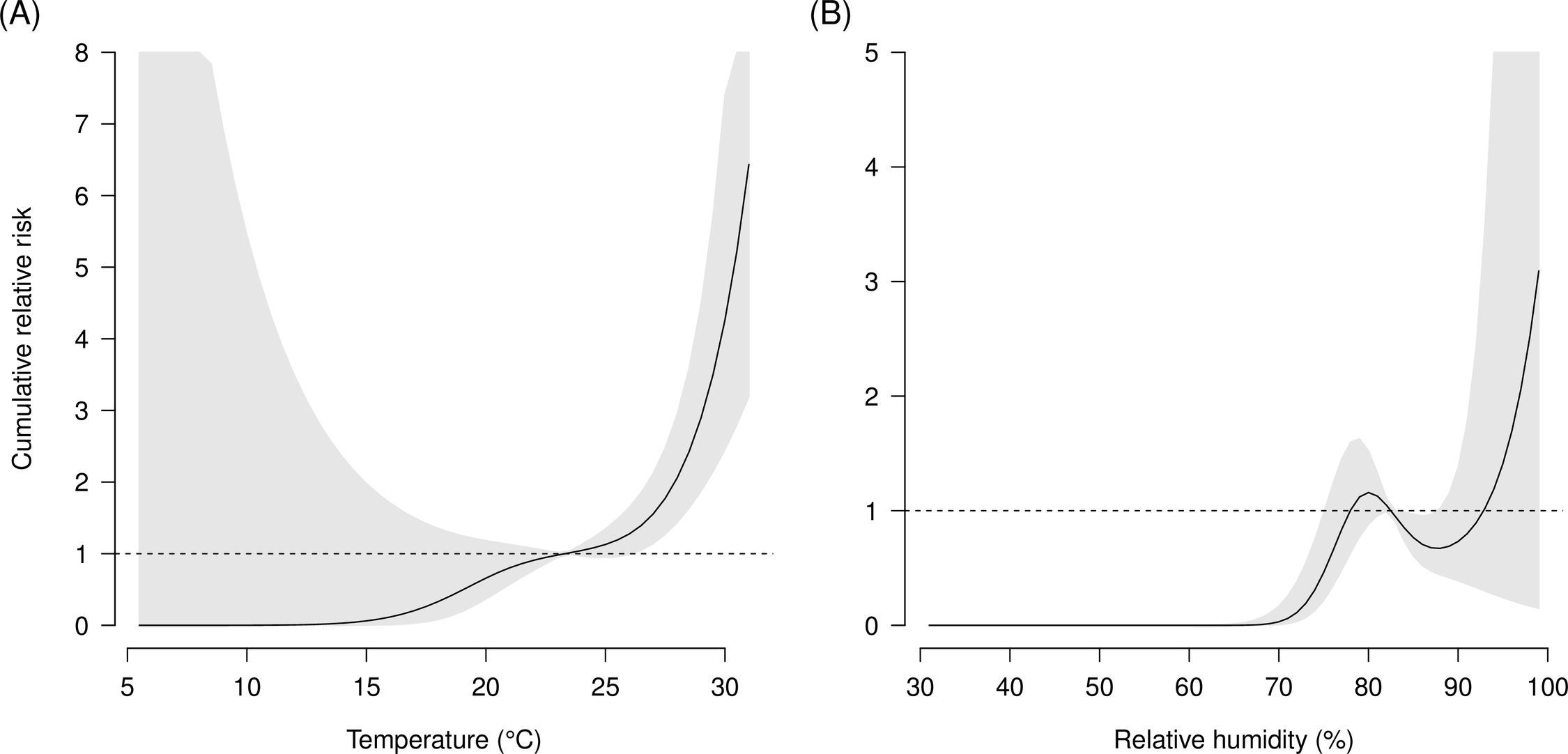

Supplement: S5 Fig — (A) Cumulative relative risk of dengue across temperature; (B) Cumulative relative risk of dengue across relative humidity. Medians of temperature and relative humidity were treated as the reference levels for the two variables. Grey regions indicate the corresponding 95% confidence intervals. (TIF) [file pntd.0008541.s005.tif]

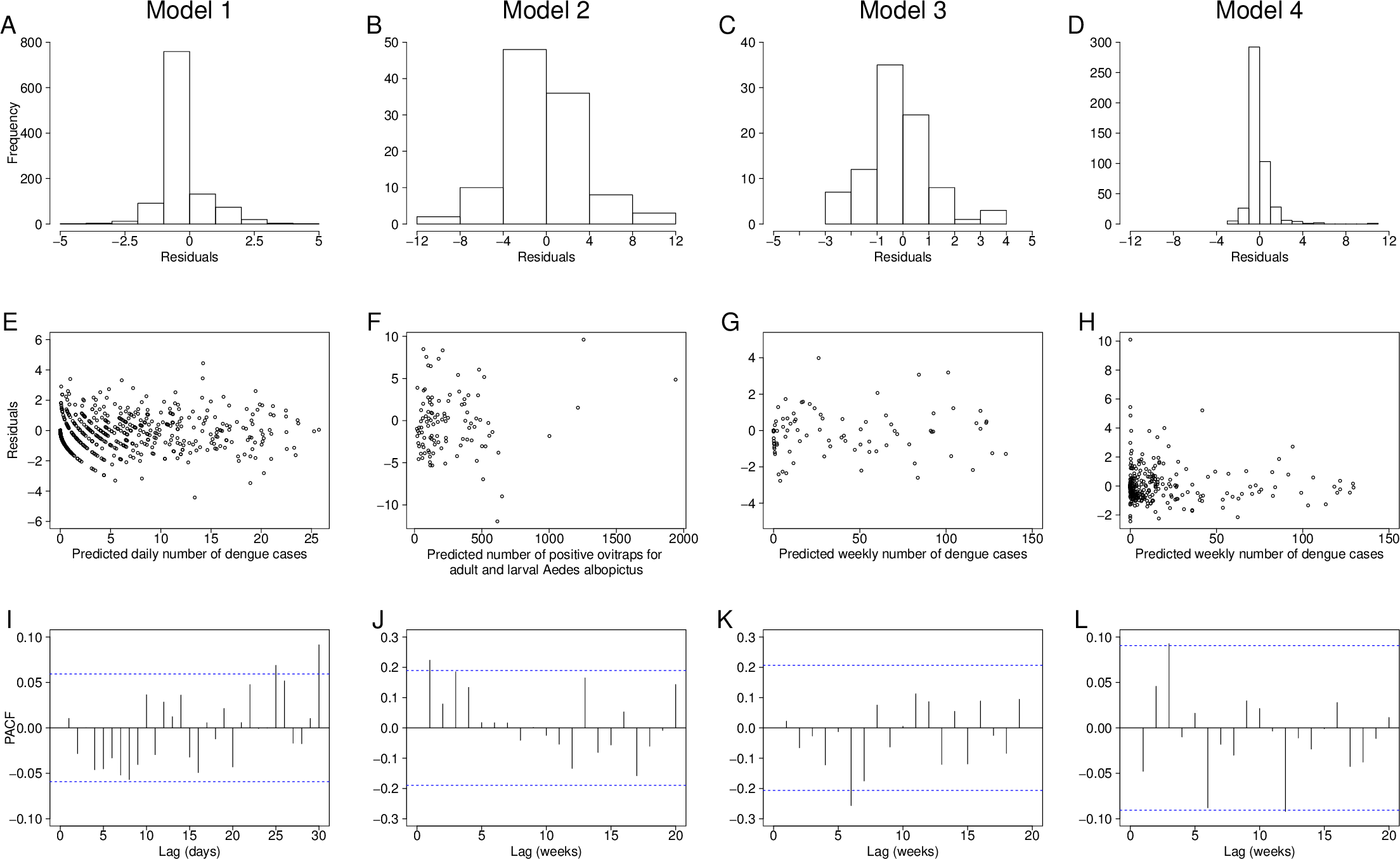

Supplement: S6 Fig — (A) Histogram of residuals of Model 1; (B) Histogram of residuals of Model 2; (C) Histogram of residuals of Model 3; (D) Histogram of residuals of Model 4; (E) Scatter plot of residuals versus predicted daily number of reported dengue cases of Model 1; (F) Scatter plot of residuals versus predicted number of positive ovitraps for adult and larval Aedes albopictus of Model 2; (G) Scatter plot of residuals versus predicted weekly number of reported dengue cases of Model 3; (H) Scatter plot of residuals versus predicted weekly number of reported dengue cases of Model 4; (I) Partial autocorrelation function (PACF) of residuals of Model 1; (J) Partial autocorrelation function of residuals of Model 2; (K) Partial autocorrelation function of residuals of Model 3; (L) Partial autocorrelation function of residuals of Model 4. (TIF) [file pntd.0008541.s006.tif]

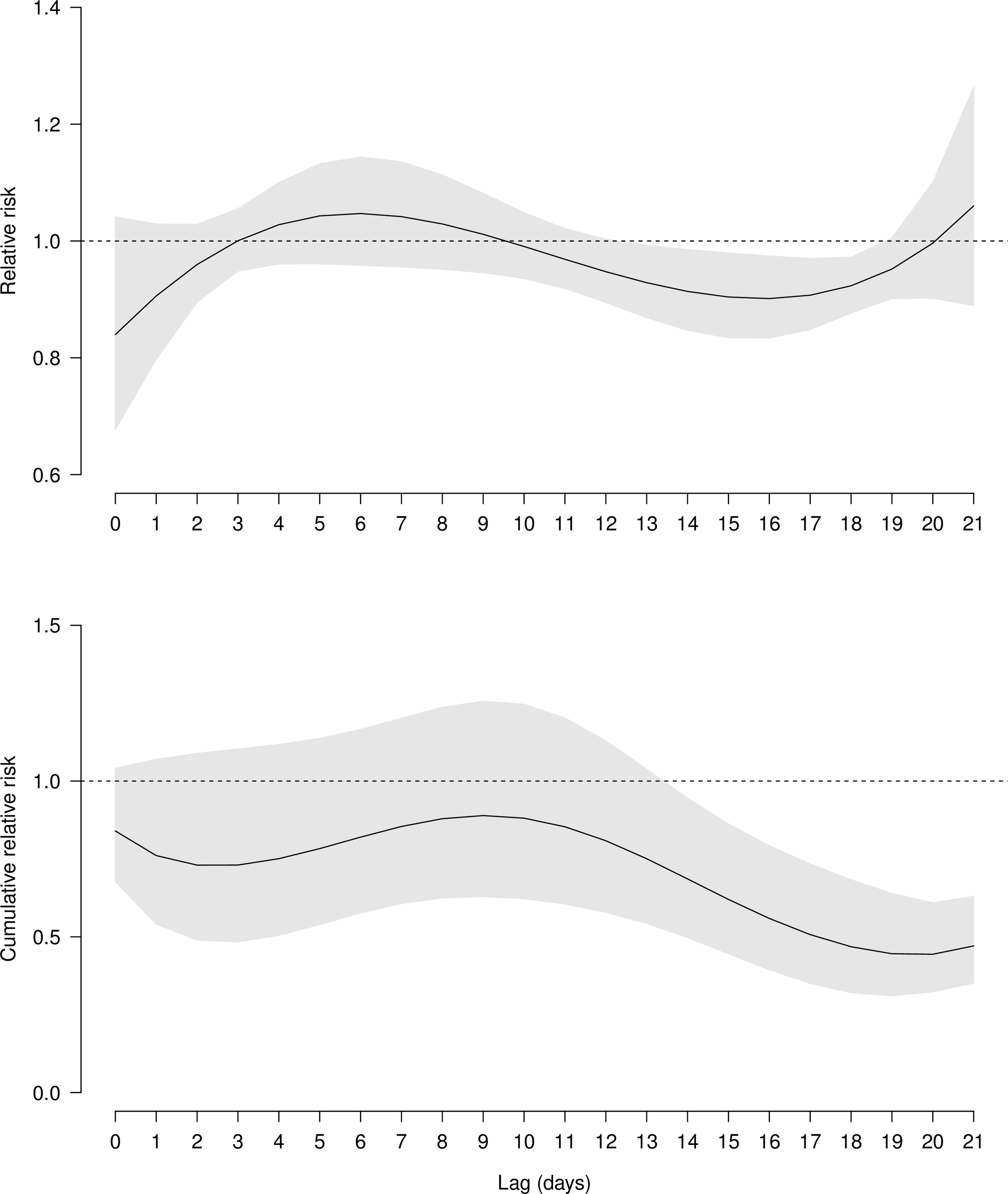

Supplement: S7 Fig — Grey regions indicate the corresponding 95% confidence intervals. (TIF) [file pntd.0008541.s007.tif]
