# Supplementary material for: The effectiveness of early start of Grade III response to dengue in Guangzhou, China: A population-based interrupted time-series study
Source: PLoS Negl Trop Dis. 2020 Aug 7;14(8):e0008541. doi: 10.1371/journal.pntd.0008541 (PMC7444500; doi:10.1371/journal.pntd.0008541)
Supplement: S1 Table — (DOCX) [file pntd.0008541.s008.docx]

**S1 Table. Values of Quasi Akaike Information Criterion (QAIC) corresponding to the models which incorporate natural cubic splines of temperature or relative humidity with different degrees of freedom.**

| Degree of freedom | QAIC | |
| --- | --- | --- |
|  | Temperature | Relative humidity |
| 1 | 2469.8 | 2465.3 |
| 2 | 2469.2 | 2446.8 |
| 3 | 2464.8 | 2458.3 |
| 4 | 2466.6 | 2456.7 |
| 5 | 2468.1 | 2452.9 |
| 6 | 2460.7 | 2451.0 |
| 7 | 2447.9 | 2443.0 |
| 8 | 2445.7 | 2447.0 |
| 9 | 2445.1 | 2447.5 |
| 10 | 2444.1 | 2447.5 |
| 11 | 2441.6 | 2446.7 |
| 12 | 2434.8 | 2448.5 |
| 13 | 2427.8 | 2448.0 |
| 14 | 2423.9 | 2448.4 |
| 15 | 2424.9 | 2445.1 |
| 16 | 2426.3 | 2444.4 |
| 17 | 2427.1 | 2445.6 |
| 18 | 2427.5 | 2448.3 |
| 19 | 2427.6 | 2447.9 |
| 20 | 2426.2 | 2444.6 |
| 21 | 2420.8 | 2444.2 |
| 22 | **2419.0** | 2446.6 |
| 23 | 2420.3 | 2448.7 |
| 24 | 2421.0 | 2450.0 |
| 25 | 2423.5 | 2449.7 |
| 26 | 2426.8 | 2449.0 |
| 27 | 2427.7 | 2448.9 |
| 28 | 2428.3 | 2445.7 |
| 29 | 2429.2 | 2444.0 |
| 30 | 2430.0 | 2444.9 |
| 31 | 2429.5 | 2443.2 |
| 32 | 2427.8 | 2442.3 |
| 33 | 2425.1 | 2440.8 |
| 34 | 2426.2 | 2440.7 |
| 35 | 2430.2 | 2437.8 |
| 36 | 2430.7 | 2430.4 |
| 37 | 2430.2 | 2422.8 |
| 38 | 2432.1 | 2417.5 |
| 39 | 2435.1 | 2413.8 |
| 40 | 2437.7 | 2409.5 |
| 41 | 2438.8 | 2408.1 |
| 42 | 2440.0 | 2406.8 |
| 43 | 2440.5 | 2405.4 |
| 44 | 2440.2 | 2402.0 |
| 45 | 2441.0 | 2400.5 |
| 46 | 2441.9 | 2399.4 |
| 47 | 2440.6 | 2394.8 |
| 48 | 2440.8 | 2393.1 |
| 49 | 2440.7 | 2391.7 |
| 50 | 2440.1 | 2388.3 |
| 51 | 2440.2 | 2386.4 |
| 52 | 2440.6 | 2385.9 |
| 53 | 2442.4 | 2387.6 |
| 54 | 2443.9 | 2388.6 |
| 55 | 2445.9 | 2389.3 |
| 56 | 2446.4 | 2392.8 |
| 57 | 2445.9 | 2393.7 |
| 58 | 2447.7 | 2393.4 |
| 59 | 2447.7 | 2392.5 |
| 60 | 2447.3 | 2390.8 |
| 61 | 2446.9 | 2390.8 |
| 62 | 2446.7 | 2391.4 |
| 63 | 2448.2 | 2390.3 |
| 64 | 2448.7 | 2390.8 |
| 65 | 2449.9 | 2388.9 |
| 66 | 2451.6 | 2386.2 |
| 67 | 2452.1 | 2382.5 |
| 68 | 2452.3 | 2377.6 |
| 69 | 2451.9 | **2373.5** |
| 70 | 2451.9 | 2378.0 |
| 71 | 2452.1 | 2381.7 |
| 72 | 2453.0 | 2388.7 |
| 73 | 2452.4 | 2383.1 |
| 74 | 2452.9 | 2380.1 |
| 75 | 2453.8 | 2383.6 |
| 76 | 2454.4 | 2385.6 |
| 77 | 2454.2 | 2390.4 |
| 78 | 2453.4 | 2393.2 |
| 79 | 2450.7 | 2388.3 |
| 80 | 2448.1 | 2386.7 |
| 81 | 2443.5 | 2380.7 |
| 82 | 2442.0 | 2381.6 |
| 83 | 2442.7 | 2379.0 |
| 84 | 2443.6 | 2380.3 |
| 85 | 2446.0 | 2381.0 |
| 86 | 2445.5 | 2378.5 |
| 87 | 2445.9 | 2380.4 |
| 88 | 2447.1 | 2378.2 |
| 89 | 2450.3 | 2382.0 |
| 90 | 2453.5 | 2391.7 |
