# Supplementary material for: The effectiveness of early start of Grade III response to dengue in Guangzhou, China: A population-based interrupted time-series study
Source: PLoS Negl Trop Dis. 2020 Aug 7;14(8):e0008541. doi: 10.1371/journal.pntd.0008541 (PMC7444500; doi:10.1371/journal.pntd.0008541)
Supplement: S2 Table — (DOCX) [file pntd.0008541.s009.docx]

**S2 Table. Values of Quasi Akaike Information Criterion (QAIC) corresponding to the models which incorporate the cross-basis of an indicator variable of the Grade III response in 2019 and time lag with different maximum lags.**

| Maximum lag | QAIC |
| --- | --- |
| 10 | 2327.0 |
| 11 | 2326.6 |
| 12 | 2324.6 |
| 13 | 2323.7 |
| 14 | 2322.5 |
| 15 | 2319.0 |
| 16 | 2318.4 |
| 17 | 2318.6 |
| 18 | 2318.6 |
| 19 | 2318.7 |
| 20 | 2318.2 |
| 21 | **2317.9** |
| 22 | 2318.5 |
| 23 | 2318.0 |
| 24 | 2318.1 |
| 25 | 2318.4 |
| 26 | 2319.6 |
| 27 | 2320.6 |
| 28 | 2321.0 |
| 29 | 2321.1 |
| 30 | 2321.2 |
| 31 | 2321.2 |
| 32 | 2321.1 |
| 33 | 2320.8 |
| 34 | 2320.8 |
| 35 | 2320.5 |
| 36 | 2320.1 |
| 37 | 2320.0 |
| 38 | 2320.5 |
| 39 | 2321.3 |
| 40 | 2321.4 |
| 41 | 2321.4 |
| 42 | 2321.2 |
| 43 | 2320.9 |
| 44 | 2320.4 |
| 45 | 2319.8 |
| 46 | 2319.0 |
| 47 | 2318.6 |
| 48 | 2318.7 |
| 49 | 2319.5 |
| 50 | 2320.6 |
| 51 | 2321.5 |
| 52 | 2322.1 |
| 53 | 2322.3 |
| 54 | 2322.3 |
| 55 | 2322.3 |
| 56 | 2322.3 |
| 57 | 2322.4 |
| 58 | 2322.3 |
| 59 | 2322.3 |
| 60 | 2322.2 |
| 61 | 2322.3 |
| 62 | 2322.4 |
| 63 | 2322.4 |
| 64 | 2322.5 |
| 65 | 2322.5 |
| 66 | 2322.4 |
| 67 | 2322.3 |
| 68 | 2322.2 |
| 69 | 2322.2 |
| 70 | 2322.2 |
| 71 | 2322.0 |
| 72 | 2322.0 |
| 73 | 2322.4 |
| 74 | 2322.5 |
| 75 | 2322.4 |
| 76 | 2322.3 |
| 77 | 2322.4 |
| 78 | 2322.5 |
| 79 | 2322.7 |
| 80 | 2322.8 |
| 81 | 2323.0 |
| 82 | 2322.9 |
| 83 | 2322.9 |
| 84 | 2323.0 |
| 85 | 2322.9 |
| 86 | 2323.1 |
| 87 | 2323.2 |
| 88 | 2323.4 |
| 89 | 2323.3 |
| 90 | 2323.4 |
