# Supplementary material for: The effectiveness of early start of Grade III response to dengue in Guangzhou, China: A population-based interrupted time-series study
Source: PLoS Negl Trop Dis. 2020 Aug 7;14(8):e0008541. doi: 10.1371/journal.pntd.0008541 (PMC7444500; doi:10.1371/journal.pntd.0008541)
Supplement: S3 Table — (DOCX) [file pntd.0008541.s010.docx]

**S3 Table. Estimates of averted number of dengue cases due to early start of Grade III response in the sensitivity analysis.**

| Model | Averted number | (95% CI) | Absolute percentage change^#^ (%) |
| --- | --- | --- | --- |
| The *df*s for calendar time* |  |  |  |
| 6 | 1,138 | (618-1,823) | 15.3 |
| 8 | 1,040 | (536-1,714) | 5.4 |
| With a dummy variable of calendar year | 989 | (481-1,645) | 0.2 |
| The *df*s for climatic variables |  |  |  |
| 4 | 1,011 | (492-1,728) | 2.4 |
| 5 | 1,024 | (461-1,799) | 3.7 |
| 6 | 903 | (338-1,689) | 8.5 |
| The minimum lag for the indicator variable of the Grade III response in 2019 (days) | 1,006 | (542-1,611) | 1.9 |
| 11 | 1,003 | (555-1,591) | 1.6 |
| 12 | 988 | (548-1,561) | 0.1 |
| 13 | 990 | (556-1,545) | 0.3 |
| 14 | 1,003 | (555-1,591) | 1.6 |
| With an inclusion of logarithm transformation of (number of imported cases + 0.5) | 953 | (493-1,561) | 3.4 |

Abbreviations: 95% CI, 95% confidence interval; *df*s, degrees of freedom.

* The results of models with a natural cubic spline function of time with 5 and 7 *df*s were not presented, since the algorithm did not converge.

^#^ Absolute percentage change equals the absolute difference in the estimates of averted number of dengue cases between sensitivity analyses and main analysis divided by the averted number of dengue cases estimated in the main analysis multiplied by 100.
