# Supplementary material for: The effectiveness of early start of Grade III response to dengue in Guangzhou, China: A population-based interrupted time-series study
Source: PLoS Negl Trop Dis. 2020 Aug 7;14(8):e0008541. doi: 10.1371/journal.pntd.0008541 (PMC7444500; doi:10.1371/journal.pntd.0008541)
Supplement: S4 Table — (DOCX) [file pntd.0008541.s011.docx]

**S4 Table. The effect of early start of Grade III response on the rate of positive ovitraps for adult and larval *Aedes albopictus*.**

| Model | *RR* | (95% CI) |
| --- | --- | --- |
| Time lags for temperature and relative humidity (weeks) |  |  |
| Temperature: 0-1; relative humidity: 0-8 | 0.62 | (0.52-0.75) |
| Temperature: 0-2; relative humidity: 0-9 | 0.64 | (0.54-0.76) |
| Temperature: 0-3; relative humidity: 0-10 | 0.65 | (0.55-0.77) |

Abbreviations: *RR*, relative risk; 95% CI, 95% confidence interval.
