# Supplementary material for: The effectiveness of early start of Grade III response to dengue in Guangzhou, China: A population-based interrupted time-series study
Source: PLoS Negl Trop Dis. 2020 Aug 7;14(8):e0008541. doi: 10.1371/journal.pntd.0008541 (PMC7444500; doi:10.1371/journal.pntd.0008541)
Supplement: S5 Table — (DOCX) [file pntd.0008541.s012.docx]

**S5 Table. The effect of the logarithm of mosquito ovitrap index (MOI) on dengue incidence.**

| Model | *RR* | (95% CI) |
| --- | --- | --- |
| The time lag for the indicator variable of the Grade III response in 2019 (weeks) |  |  |
| 2 | 1.37 | (1.00-1.88) |
| The time lag between the logarithm of MOI and dengue incidence (weeks) |  |  |
| 2-3 | 1.33 | (1.02-1.74) |
| 3 | 1.24 | (1.00-1.54) |

Abbreviations: *RR*, relative risk; 95% CI, 95% confidence interval.
