# Supplementary material for: The effectiveness of early start of Grade III response to dengue in Guangzhou, China: A population-based interrupted time-series study
Source: PLoS Negl Trop Dis. 2020 Aug 7;14(8):e0008541. doi: 10.1371/journal.pntd.0008541 (PMC7444500; doi:10.1371/journal.pntd.0008541)
Supplement: S1 Text — (DOCX) [file pntd.0008541.s013.docx]

## S1 Text. Events related to dengue of six grades and the corresponding responses

# Grade VI events related to dengue

The Grade VI events related to dengue are defined as (1) Breteau index (BI, the number of containers detected positive for larval *Aedes albopictus* per 100 households which were sampled)>20; or (2) standard space index (SSI, the number of containers detected positive for larval *Aedes albopictus* per 100 outdoor standard spaces (i.e. 15 square meters))>2; or (3) mosquito ovitrap index (MOI, the number of positive ovitraps for adult and larval *Aedes albopictus* per 100 traps which were retrieved)>20; or (4) adult density index (ADI, the number of adult female mosquitoes collected per person per hour)>10; and (5) no dengue case being reported in a street or town.

# Grade VI response

The objectives of Grade VI response include (1) controlling the mosquito vector density and (2) preventing dengue fever outbreaks. A subdistrict government has the responsibilities to (1) kill adult mosquitoes in the places where mosquito vector density exceeds the safety threshold. ADI and MOI should be reduced to the safety level within three and seven days, respectively; (2) eliminate mosquito breeding ground until BI and SSI are reduced to the safety level within seven days; (3) promote the information on the health risk of being exposed to mosquito vectors and guide residents how to kill adult mosquitoes and remove mosquito breeding sites; (4) report to local government and Department of Health of the corresponding district on the work of dengue prevention and control.

Department of Health of a district is required to (1) evaluate the work of the control of mosquito vectors in the streets or towns and conduct surveillance on mosquito vector density if necessary; (2) provide technical guidance on how to control mosquito vectors for the streets or towns; (3) enhance control of mosquito vectors in high-risk areas; (4) supervise and inspect the responses of relevant streets and towns and report to local government of the district. Department of Urban Management of a district should organize a group of people to keep the district clean and remove stagnant water. Department of Health of the city is responsible for supervising, inspecting and reporting the work of control of dengue in relevant districts.

# Grade V events related to dengue

The Grade V events related to dengue are defined as (1) imported cases occurring in a district and BI>5 in the core area; or (2) local cases being reported in a district.

# Grade V response

The objectives of Grade V response include (1) performing well in case management; (2) working properly in anti-mosquito isolation; (3) reducing mosquito vector density; (4) preventing the spread of dengue fever outbreaks. In addition to implementing Grade VI response, a subdistrict government has the responsibilities to (1) screen dengue cases and arrange hospitalization for the patients; (2) strengthen the promotion of information on the health risk of being exposed to mosquito vectors, remove mosquito breeding grounds in warning zones and monitoring areas according to the instructions proposed by Guangzhou Center for Disease Control and Prevention (GZ CDC) and kill adult mosquitoes quickly if necessary; (3) carry out daily report on the response for the street or town.

Department of Health of a district is required to (1) organize and coordinate relevant departments to implement various measures for dengue control, enhance the surveillance for dengue, risk assessment and report of outbreaks; (2) organize people to conduct epidemiological investigation, manage and provide healthcare services for dengue cases, and direct dengue control in epidemic spots; (3) supervise the work of reducing mosquito vector density, requiring that mosquito vector density should be reduced to safety level for core and warning zones and to the low-risk level in other places; (4) launch a series of activities to remove mosquito breeding sites; (5) intensify mosquito surveillance, assess the effects of mosquito control measures, enhance risk assessment and early warning processes, and report questions and suggestions to local government and relevant departments. Department of Urban Management of a district should (1) strengthen supervision and law enforcement, making sure that mosquito control measures can be implemented smoothly; (2) keep the environment clean, especially eliminate mosquito breeding grounds. Department of Health of the city should report the responses to dengue to provincial health authorities, review and give feedback to the results of samples collected from districts. The sample of the first local dengue case should be sent to provincial CDC to be confirmed within two working days.

# Grade IV events related to dengue

The Grade IV events related to dengue are defined as (1) the weekly number of dengue cases reported in a district reaching five but being less than 10; or (2) local cases being reported in more than three streets or towns in a district within one week; or (3) an outbreak occurring in a district.

# Grade IV response

The objectives of Grade IV response include (1) stamping out the outbreak promptly; (2) preventing the spread of dengue. In addition to implementing Grade V response, a subdistrict government has the responsibilities to (1) coordinate implementation of the measures for dengue prevention and control; (2) urge staffs in different institutions, such as those working in parks, schools and hospitals to set up a cleaning team for removing mosquito breeding grounds extensively and killing adult mosquitoes effectively; (3) encourage residents to eliminate mosquito breeding grounds and organize a group of people for inspection; (4) strengthen the promotion of the knowledge of dengue prevention and control.

Department of Health authority of a district is required to (1) suggest the local government to start the response to dengue according to the results of risk assessment and strengthen joint prevention and control of dengue; (2) gather the professional teams for emergency mosquito control in a district and subordinate streets or towns to reduce mosquito vector density in key areas; (3) launch a series of activities to kill adult mosquitoes, remove mosquito breeding grounds; (4) implement emergency case surveillance; (5) perform risk assessment once a week and report the information of dengue epidemic and effectiveness of measures for dengue control to health authorities and local government of the city and other relevant departments.

Department of Urban Management of a district should increase the frequency of cleaning especially in the areas where dengue outbreaks occur. Department of Housing and construction of a district is required to supervise the mosquito-control work in construction sites and residential areas. Department of Education of a district should organize activities that aim at reducing the exposure to mosquito vectors in schools and introduce the knowledge of how to prevent dengue, such as eliminating mosquito breeding grounds, into the courses. Department of Forests of a district should implement measures for mosquito prevention and control in parks, scenic areas, urban greenbelts, disseminate knowledge about dengue, help for emergency treatment of dengue. Department of Water Affairs of a district should improve the dredging work, preventing mosquito breeding. Department of Tourism of a district should disseminate the information on how to prevent and control dengue among those who plan to travel abroad, encourage hotels, restaurants, travel agencies, scenic areas to implement vector control measures and to help for emergency treatment of dengue. Department of Finance of a district should make sure the funding for dengue prevention and control and for treatment of patients is sufficient. Department of Publicity of a district should coordinate the report of the work of dengue prevention and control and the promotion of knowledge of how to prevent and control dengue. Relevant departments at city level should supervise the work of dengue prevention and control at district level, help for the implementation of the work and establish a system for weekly or daily report if necessary.

# Grade III events related to dengue

The Grade III events related to dengue are defined as (1) the weekly number of dengue cases reported in a district reaching 10 but being less than 100; or (2) the Grade IV events related to dengue (i.e. [a] the weekly number of dengue cases reported in a district reaching five but being less than 10; or [b] local cases being reported in more than three streets or towns in a district within one week; or [c] an outbreak occurring in a district) occurring in two districts within 14 days; or (3) the total number of dengue cases reaching the double of the average level in the past five years.

# Grade III response

The aims of the Grade III response are quickly controlling dengue outbreak and preventing rapid spread of dengue in the population. In addition to implementing Grade IV response, Office of Public Health Emergency Response of the city should organize the work of dengue prevention and control for district government and relevant departments at city level, ask for help from provincial government and relevant departments at provincial level. Department of Health of the city should (1) recommend local government of the city to initiate Grade III response and strengthen joint prevention and control of dengue; (2) organize care for dengue patients, resources, mosquito and case surveillance, assessment of effectiveness of outbreak management and measures of mosquito control, promotion of protective measures, mosquito control activities, supervise the work of each district; (3) ask for technical support from health authorities at provincial level.

Department of Urban Management of the city should organize the work of cleaning the environment, especially removing mosquito breeding grounds. Other departments at city level should implement the measures for dengue prevention and control, supervise the work of each district, and report the work to Office of Public Health Emergency Response of the city once a week. Local governments at district level should guarantee that the measures of dengue prevention and control are implemented smoothly and report the work to Office of Public Health Emergency Response of the city once a week.

# Grade II events related to dengue

The Grade II events related to dengue are defined as (1) the weekly number of dengue cases reported in a district reaching 100; or (2) the epidemic spreading in more than two district within one week and the total number of dengue cases exceeding the double of the average level in the past five years. Provincial government further decides whether the events are Grade II events related to dengue.

# Grade I events related to dengue

The Grade I events related to dengue are defined as the Grade II events related to dengue occurring in two or more than two provinces including Guangdong province. National government further decides whether the events are Grade I events related to dengue.

# Grade II and I responses

In addition to implementing Grade III response, local government of the city and relevant departments should implement the measures for dengue prevention and control following the arrangement formulated by national and provincial governments [1].

# Reference

1. Guangzhou Municipal Health and Family Planning Commission. Preparedness and response plan for dengue fever (2018). 2018; [cited June 25, 2020]. Available from: <http://www.gzcdc.org.cn/uploads/download/20191107/%E5%B9%BF%E5%B7%9E%E5%B8%82%E7%99%BB%E9%9D%A9%E7%83%AD%E7%96%AB%E6%83%85%E5%BA%94%E6%80%A5%E9%A2%84%E6%A1%88%EF%BC%882018%E5%B9%B4%E7%89%88%EF%BC%89.pdf>.
