# Supplementary material for: The effectiveness of early start of Grade III response to dengue in Guangzhou, China: A population-based interrupted time-series study
Source: PLoS Negl Trop Dis. 2020 Aug 7;14(8):e0008541. doi: 10.1371/journal.pntd.0008541 (PMC7444500; doi:10.1371/journal.pntd.0008541)
Supplement: S2 Text — (DOCX) [file pntd.0008541.s014.docx]

## S2 Text. The safety level of mosquito vector density

The safety level of mosquito vector density means (1) adult density index (ADI, the number of adult female mosquitoes collected per person per hour)<2; (2) mosquito ovitrap index (MOI, the number of positive ovitraps for adult and larval *Aedes albopictus* per 100 traps which were retrieved)<5; (3) Breteau index (BI, the number of containers detected positive for larval *Aedes albopictus* per 100 households which were sampled)<5; and (4) standard space index (SSI, the number of containers detected positive for larval *Aedes albopictus* per 100 outdoor standard spaces [i.e. 15 square meters])<1.
