# Supplementary material for: The effectiveness of early start of Grade III response to dengue in Guangzhou, China: A population-based interrupted time-series study
Source: PLoS Negl Trop Dis. 2020 Aug 7;14(8):e0008541. doi: 10.1371/journal.pntd.0008541 (PMC7444500; doi:10.1371/journal.pntd.0008541)
Supplement: S3 Text — (DOCX) [file pntd.0008541.s015.docx]

## S3 Text. Model 1

We compared the effect of Grade III response commencing in August 2019 on dengue incidence with the counterfactual scenario that the Grade III response started in September as usual by associating the indicator variable of the Grade III response in 2019 and daily incidence of dengue using a quasi-Poisson regression model [1]. The model evaluating the effect of early start of Grade III response on dengue incidence (Model 1) was as follows:

$$\begin{matrix} \log(E[D_{t}])=\alpha_{D}+offset(\log[Pop_{t}])+ns(Day_{t},df=4)+\gamma_{D}Year_{t} \\ \begin{matrix} & & & \begin{matrix} & +\theta_{Temp}Temp_{t,0-22}+\theta_{Hum}Hum_{t,0-69}+\beta_{D}Int_{t,10-21} \end{matrix} \end{matrix}\begin{matrix} \begin{matrix} & & & \begin{matrix} & & & \begin{matrix} & \end{matrix} \end{matrix} \end{matrix} & (1) \end{matrix} \end{matrix}$$

where *t* is sequential days from January 1, 2017 to December 31, 2019 (*t* = 1, 2, 3, …, 1,095); *D_t_* and *Pop_t_* are daily number of dengue cases and population at the time point *t*, respectively. We included the logarithm transformation of population into the model and constrained the corresponding regression coefficient to be one. A natural cubic spline function *ns*(.) with four degrees of freedom (*df*s) was used for the calendar day in each year (*Day_t_* = 1, 2, 3, …, 365) to control for the seasonality of dengue incidence. In addition, a linear function of calendar year was also included. A distributed lag non-linear model (DLNM) can depict the delayed effect of a variable flexibly by presenting the effect in the space of variable and lag dimension simultaneously [2]. In the present study, the cross-basis produced by applying the DLNM to candidate meteorological variables, including daily mean temperature and relative humidity, and a categorical variable of rainfall, was used to describe their impacts on dengue incidence. We used a natural cubic spline with three *df*s for climatic variables and time lags [3]. To adequately capture the delayed effects, the time lags were set to be at most 90 days, considering the lifespan of *Aedes* mosquitoes, intrinsic incubation period (IIP), and the time delay in the reporting of dengue cases [3-5]. Since the information of relative humidity and rainfall overlapped, we separately examined one of them in the model. The climatic variables and time lags were selected based on the Quasi Akaike Information Criterion (QAIC). The metrics obtained by applying the DLNM to temperature (*Temp_t_*_,0-22_) and relative humidity (*Hum_t_*_,0-69_), with maximum lags of 22 and 69 days, were included in Model 1. Then, the cross-basis of an indicator variable of the Grade III response in 2019 (0: before August 9, 2019; 1: otherwise) and time lag was included in the model. The cross-basis can indicate the additional effect of early start of Grade III response, since the Grade III response started in September in 2017 and 2018, but in August in 2019. We used a degree two polynomial for time lag and constrained it to be at least 10 days, considering the IIP and time delay from illness onset to reporting cases. In addition, we assumed that the impact of early start of Grade III response lasted for at most 90 days and selected the time lag according to QAIC. The metric obtained by applying the DLNM to the indicator variable of the Grade III response in 2019, with a lag of 10-21 days (*Int_t,_*_10-21_), was included in Model 1.

## References

1. Liyanage P, Rocklov J, Tissera H, Palihawadana P, Wilder-Smith A, Tozan Y. Evaluation of intensified dengue control measures with interrupted time series analysis in the Panadura Medical Officer of Health division in Sri Lanka: a case study and cost-effectiveness analysis. Lancet Planet Health. 2019;3(5):e211-e8.

2. Gasparrini A, Armstrong B, Kenward MG. Distributed lag non-linear models. Stat Med. 2010;29(21):2224-34.

3. Cheong YL, Burkart K, Leitao PJ, Lakes T. Assessing weather effects on dengue disease in Malaysia. Int J Environ Res Public Health. 2013;10(12):6319-34.

4. Maimusa HA, Ahmad AH, Kassim NF, Rahim J. Age-stage, two-sex life table characteristics of Aedes albopictus and Aedes Aegypti in Penang Island, Malaysia. J Am Mosq Control Assoc. 2016;32(1):1-11.

5. Chan M, Johansson MA. The incubation periods of Dengue viruses. PLoS One. 2012;7(11):e50972.
