# Supplementary material for: The effectiveness of early start of Grade III response to dengue in Guangzhou, China: A population-based interrupted time-series study
Source: PLoS Negl Trop Dis. 2020 Aug 7;14(8):e0008541. doi: 10.1371/journal.pntd.0008541 (PMC7444500; doi:10.1371/journal.pntd.0008541)
Supplement: S4 Text — (DOCX) [file pntd.0008541.s016.docx]

## S4 Text. Model 2 and Model 3

We hypothesized that the Grade III response started one month ahead in August could influence dengue incidence through affecting mosquito vector density (i.e., mosquito vector density could be a mediator between the change in the start time of Grade III response and dengue incidence). Quasi-Poisson regression models were applied to evaluate the effect of early start of Grade III response on the rate of positive ovitraps for adult and larval *Aedes albopictus* (Model 2) and to associate the indicator variable of the Grade III response in 2019, logarithm transformation of MOI, and weekly number of dengue cases (Model 3). Model 2 and Model 3 were as follows:

$$\begin{matrix} \log(E[Pos_{tw}])=\alpha+offset(\log[Trap_{tw}])+ns(Week_{tw},df=4) \\ \begin{matrix} & & & \begin{matrix} & \begin{matrix} +\gamma Year_{tw}+ns(Temp_{tw},df=3)+ns(Hum\text{\_0-7}_{tw},df=3) \\ +\beta Int_{tw}\begin{matrix} & & & \begin{matrix} & & & \begin{matrix} & & & \begin{matrix} & & & \begin{matrix} & & \begin{matrix} & & & \begin{matrix} & \end{matrix} \end{matrix} & (2) \end{matrix} \end{matrix} \end{matrix} \end{matrix} \end{matrix} \end{matrix} \end{matrix} \end{matrix} \end{matrix}$$

$$\begin{matrix} \log(E[D_{tw}])=\alpha_{Dw}+offset(\log[Pop_{tw}])+ns(Week_{tw},df=4) \\ \begin{matrix} & & & \begin{matrix} & \begin{matrix} +\gamma_{Dw}Year_{tw}+ns(Temp\text{\_0-3}_{tw},df=3) \\ +ns(Hum\text{\_0-10}_{tw},df=3) \\ +\delta\log(MOI\text{\_1-3}_{tw})+\beta_{Dw}Int_{tw-3}\begin{matrix} & & & \begin{matrix} & & & \begin{matrix} & & & \begin{matrix} & & & (3) \end{matrix} \end{matrix} \end{matrix} \end{matrix} \end{matrix} \end{matrix} \end{matrix} \end{matrix}$$

where *tw* is sequential weeks from 1 to 156; *Pos_tw_* and *Trap_tw_* are the number of positive ovitraps for adult and larval *Aedes albopictus* and total number of traps at the time point *tw*, respectively; *Week_tw_* represents the calendar week in each year (*Week_tw_* = 1, 2, 3, …, 52); *D_tw_* and *Pop_tw_* are the number of dengue cases and population at the time point *tw*, respectively. According to the results of Model 1, moving averages of 0-3 and 0-10 weeks were applied to temperature and relative humidity for Model 3, respectively.

The time lags between climatic variables and the rate of positive ovitraps for adult and larval *Aedes albopictus* in Model 2 and the lag between the logarithm of MOI and dengue incidence in Model 3 were selected jointly, assuming that climatic factors could influence dengue incidence through mosquito vector density. We examined six combinations of time lags (S3 Fig), considering that (1) a time lag of 22 days was selected for the effect of temperature on dengue incidence in Model 1; (2) there should be a time delay between mosquito vector density and dengue incidence, considering the intrinsic incubation period (IIP) and the time lag between illness onset and case report.
