# Supplementary material for: The effectiveness of early start of Grade III response to dengue in Guangzhou, China: A population-based interrupted time-series study
Source: PLoS Negl Trop Dis. 2020 Aug 7;14(8):e0008541. doi: 10.1371/journal.pntd.0008541 (PMC7444500; doi:10.1371/journal.pntd.0008541)
Supplement: S5 Text — (DOCX) [file pntd.0008541.s017.docx]

## S5 Text. Sensitivity analysis

We conducted sensitivity analysis to (1) assess the cumulative relative risk (*RR*) of dengue over 0-21 days associated with one-month early start of Grade III response, checking whether the effect of one-month early start of Grade III response began within 10 days; (2) check the robustness of the estimate of total number of dengue cases avoided due to early start of Grade III response in 2019 by: (2a) changing the *df* for time in the model to 6 and 8 (results for *df* =5 and *df* =7 were not reported since the algorithm did not converge); (2b) including into Model 1 the dummy variable instead of linear function of calendar year; (2c) increasing the *df*s for the natural cubic spline functions for meteorological factors to 4-6; (2d) changing the minimum time lag between the early start of Grade III response and dengue incidence to 11-14 days; (2e) including the logarithm transformation of (number of imported cases + 0.5) in the model (3) check the robustness of the estimate of *RR* of the positive rate due to early start of Grade III response by changing the time lags to 0-1, 0-2, 0-3 weeks for temperature and to 0-8, 0-9, 0-10 weeks for relative humidity; and (4) check the robustness of the estimate of *RR* of dengue due to the change in the logarithm of MOI, after controlling for the effect of early start of Grade III response by (4a) changing the time lag between the early start of Grade III response and dengue incidence to two weeks; (4b) changing the time lag between the logarithm of MOI and weekly dengue incidence to 2-3 and 3 weeks.

Sensitivity analysis indicated that (1) the *RR* of dengue due to early start of Grade III response over 0-9 days fluctuated around one and was statistically non-significant (S7 Fig); (2) the estimates of averted number of dengue cases did not vary substantially with the specification of some parameters in models, with absolute percentage changes ranging between 0.1% and 15.3% (S3 Table) ; (3) the effect of early start of Grade III response on the positive rate of ovitraps remained stable (S4 Table) ; and (4) the magnitude of the effect of MOI on dengue incidence was also robust to the specification of parameters (S5 Table).
