# Supplementary material for: The effectiveness of early start of Grade III response to dengue in Guangzhou, China: A population-based interrupted time-series study
Source: PLoS Negl Trop Dis. 2020 Aug 7;14(8):e0008541. doi: 10.1371/journal.pntd.0008541 (PMC7444500; doi:10.1371/journal.pntd.0008541)
Supplement: Attachment — Submitted filename: Responses to Reviewers comments.docx [file pntd.0008541.s018.docx]

**Responses to reviewers' comments**

# Reviewer #1:

**R1.1.** Up to date, dengue is not endemic in China. So dengue cases consist of imported cases and local cases in China. I recommend the authors divide dengue cases into imported cases and local cases prior to analysis. Grade III response mainly reduce the number of local cases. Of note, the number of local cases not only influenced by Grade III response but also influenced by the number of imported cases.

**[Response] We thank reviewer for the suggestion. Among the total of 3,871 dengue cases in Guangzhou over the study period, there were 417 (10.8%) imported cases and 3,454 (89.2%) local cases. We assessed the impact of early start of Grade III response on local dengue incidence (page 10, line 183; page 11, lines 212, 217). In addition, we fitted a model which included the information on the number of imported cases in the sensitivity analysis in the revision. Results suggested that the averted numbers of dengue cases due to early start of Grade III response estimated with and without adjustment for number of imported cases were similar (953 vs 987) (S3 Table).**

**R1.2.** Grade III response can reduce the number of dengue cases. First, Grade III response can reduce mosquito density which can indirect reduce the transmission of dengue viruses. Second, Grade III response may change the activities of humans or improve human’s ability of preventing dengue which direct reduce the number of dengue. SEM should be conducted to identify direct and indirect impacts of Grade III response.

**[Response] We agree that the results would be more informative if the estimates of direct and indirect impacts of early start of Grade III response can be provided. SEM has been extensively applied to cross-sectional data to examine whether a variable is a mediator and quantify direct and indirect impacts of a variable. However, in our study, the potential mediator effect of mosquito vector density was determined based on time-series data. The commonly used Baron and Kenny’s approach was applied to conduct the mediation analysis through fitting Model 1-Model 3 [1, 2] (S2 Fig).**

**S2 Fig.** Models used to test whether mosquito vector density was a mediator between early start of Grade III response and dengue incidence.

**In details, we estimated the total effect of early start of Grade III response on dengue incidence based on Model 1 and the result suggested that early start of Grade III response mitigated the dengue burden (relative risk [*RR*]: 0.54, 95% confidence interval [CI]: 0.43-0.70)**. **We found that mosquito vector density could be a mediator of early start of Grade III response and dengue incidence, given the results of Model 1-Model 3 satisfied the prerequisites for establishing mediation proposed by Baron and Kenny, i.e. (1) the independent variable (early start of Grade III response) is a predictor of the dependent variable (dengue incidence) (Model 1); (2) the independent variable has an effect on the mediator (mosquito vector density) (Model 2, *RR*: 0.64, 95% CI: 0.53-0.77); (3) the mediator is a predictor of the dependent variable after controlling for the effect of the independent variable (Model 3, *RR*: 1.36, 95% CI: 1.01-1.84). In addition, the results of Model 3 revealed that the effect of early start of Grade III response on dengue incidence was weakened while still statistically significant after controlling for the effect of mosquito vector density (*RR*: 0.68, 95% CI: 0.52-0.90) (Table 1). The finding, as the reviewer mentioned, suggested that “Early start of Grade III response may change the activities of humans or improve human’s ability of preventing dengue which direct reduce the number of dengue”. That is, mosquito vector density is a mediator that partially mediated the relationship between early start of Grade III response and dengue incidence.**

**We did not perform SEM analysis, because it is difficult to estimate the direct and indirect lagged effects for the non-Gaussian time series data using SEM currently. To our knowledge, some methods based on SEM were proposed for the evaluation of longitudinal mediation. Among these methods, cross-lagged panel model (CLPM), latent growth curve model (LGM) and latent change score (LCS) focus on how the individual differences in independent variable predict dependent variable through mediator, but not treat mediation as a within-person process [3]. Therefore, these methods are not applicable to the time-series data used in our study. The distributed-lag linear structural equation model (DLSEM) can be used to depict the lagged effect of independent variable. However, the method has not been generalized to non-Gaussian data [4, 5]. Thus, the method is not applicable to the count data in our study. We discussed this in the section of limitation in the revision (page 21, lines 405-410).**

**References:**

1.	Baron RM, Kenny DA. The moderator-mediator variable distinction in social psychological research: conceptual, strategic, and statistical considerations. J Pers Soc Psychol. 1986;51(6):1173-82. Epub 1986/12/01. doi: 10.1037//0022-3514.51.6.1173. PubMed PMID: 3806354.

2.	MacKinnon DP, Fairchild AJ, Fritz MS. Mediation analysis. Annu Rev Psychol. 2007;58:593-614. doi: 10.1146/annurev.psych.58.110405.085542. PubMed PMID: 16968208.

3.	Preacher KJ. Advances in mediation analysis: a survey and synthesis of new developments. (1545-2085 (Electronic)).

4.	Magrini A. Distributed-lag Linear Structural Equation Models in R: the dlsem Package. Austrian Journal of Statistics. 2019;48(2):14-42. doi: 10.17713/ajs.v48i2.777.

5.	Magrini, A., Bartolini, F., Coli, A. et al. A structural equation model to assess the impact of agricultural research expenditure on multiple dimensions. Qual Quant 53, 2063–2080 (2019). <https://doi.org/10.1007/s11135-019-00855-z>.

# Reviewer #2:

**R2.1.** The response for the dengue includes six grades, which grade is chosen based on the severity of event. The question is whether the grade will be upgraded to a higher grade when the cases are increasing during an outbreak. For example, the cases were relatively lower when Grade III was launched in 2019, whether the intervention was strengthened with cases increasing. As the authors mentioned in the paper, the responses are comprised mainly of vector surveillance, vector control, raising public awareness etc., which are not quantified measurements, how to sustain the consistency of the intervention during an outbreak, and the comparability among 2017, 2018 and 2019.

**[Response] We thank reviewer for the insightful comments. The grade will be upgraded to a higher grade when the cases are increasing during an outbreak. For example, the Grade III response will be updated to Grade II response when the number of new cases in a district within one week exceeded 100, while the number of new cases for triggering Grade III response is larger than 10 and less than 100 (S1 File). During 2017-2019, the local government implemented Grade III response, while higher-grade responses (i.e. Grade I and II responses) were not implemented, since the dengue epidemics in the three years were not severe enough to trigger these responses.**

**We apologized that we did not explain the differences of the responses of the six grades clearly. In fact, the six grades of responses from grade VI to grade I correspond to increasing dengue events as mentioned in the revised manuscript. The intervention is intensified, additional works are required to be conducted, and more human and financial resources are invested when the response is upgraded, especially when the Grade III response and higher responses are commenced. Local government dominates the work of dengue control once Grade III-I responses start. The direct supervision of senior officials guarantees that much more efforts are taken on the control of dengue. We described the definitions of dengue events of six grades and the corresponding responses in the revision (S1 File).**

**We admitted that the intensity of intervention for dengue control might change, but it should be within a certain range for a specific grade of response and distinguished from those for other grades [6], although it is extremely difficult to quantify the intervention intensity. We mentioned it as a limitation in the revision: “Next, the intensity of intervention for dengue control might fluctuate during the period of Grade III response. However, the intervention intensity of Grade III response should be within a certain range and should be significantly stronger than that of Grade VI-IV responses given the high degree of government involvement [6]. Since it is extremely difficult to quantify the intervention intensity, we only used a dummy variable indicating the period of Grade III response in 2009 in the model to examine the average effect of early start of Grade III response on dengue incidence.” (pages 20-21, lines 398-405).**

**Reference:**

6.	Guangzhou Municipal Health and Family Planning Commission. Preparedness and response plan for dengue fever (2018). 2018; [cited June 25, 2020]. Available from: <http://www.gzcdc.org.cn/uploads/download/20191107/%E5%B9%BF%E5%B7%9E%E5%B8%82%E7%99%BB%E9%9D%A9%E7%83%AD%E7%96%AB%E6%83%85%E5%BA%94%E6%80%A5%E9%A2%84%E6%A1%88%EF%BC%882018%E5%B9%B4%E7%89%88%EF%BC%89.pdf>.

**R2.2.** The study indicated mosquito density was a mediator of intensified dengue control measures, climatic factors and dengue incidence. Therefore, it may be not appropriate that mosquito density, and climatic factors and dengue control measures were recognized as the independent variables simultaneously to interpret dengue incidence as in model 3, but path analysis or structural equation model can.

**[Response] In our study, we examined whether mosquito vector density was a mediator between early start of Grade III response and dengue incidence according to the prerequisites proposed by Baron and Kenny: (1) the independent variable (early start of Grade III response) is a predictor of the dependent variable (dengue incidence) (Model 1); (2) the independent variable has an effect on the mediator (mosquito vector density) (Model 2); (3) the mediator is a predictor of the dependent variable after controlling for the effect of the independent variable (Model 3) [1, 2] (S2 Fig).**

**S2 Fig.** Models used to test whether mosquito vector density was a mediator between early start of Grade III response and dengue incidence.

**We did not perform structural equation modeling (SEM) analysis, because it is difficult to estimate the direct and indirect lagged effects for the non-Gaussian time series data using SEM currently. To our knowledge, some methods based on SEM were proposed for the evaluation of longitudinal mediation. Among these methods, cross-lagged panel model (CLPM), latent growth curve model (LGM) and latent change score (LCS) focus on how the individual differences in independent variable predict dependent variable through mediator, but not treat mediation as a within-person process [3]. Therefore, these methods are not applicable to the time-series data used in our study. The distributed-lag linear structural equation model (DLSEM) can be used to depict the lagged effect of independent variable. However, the method has not been generalized to non-Gaussian data [4, 5]. Thus, the method is not applicable to the count data in our study. We discussed this in the section of limitation in the revision (page 21, lines 405-410).**

**References:**

1.	Baron RM, Kenny DA. The moderator-mediator variable distinction in social psychological research: conceptual, strategic, and statistical considerations. J Pers Soc Psychol. 1986;51(6):1173-82. Epub 1986/12/01. doi: 10.1037//0022-3514.51.6.1173. PubMed PMID: 3806354.

2.	MacKinnon DP, Fairchild AJ, Fritz MS. Mediation analysis. Annu Rev Psychol. 2007;58:593-614. doi: 10.1146/annurev.psych.58.110405.085542. PubMed PMID: 16968208.

3.	Preacher KJ. Advances in mediation analysis: a survey and synthesis of new developments. (1545-2085 (Electronic)).

4.	Magrini A. Distributed-lag Linear Structural Equation Models in R: the dlsem Package. Austrian Journal of Statistics. 2019;48(2):14-42. doi: 10.17713/ajs.v48i2.777.

5.	Magrini, A., Bartolini, F., Coli, A. et al. A structural equation model to assess the impact of agricultural research expenditure on multiple dimensions. Qual Quant 53, 2063–2080 (2019). <https://doi.org/10.1007/s11135-019-00855-z>.

**R2.3.** It’s known that rainfall influences the mosquito density and then influences dengue incidence. I notice that rainfall was not included into the model based on the study’s statistical strategy. Daily data on rainfall were downloaded, why rainfall was arbitrarily categorized into three levels not as the continuous variable was included in the model.

**[Response] Rainfall could influence dengue incidence and was considered as a three-category variable in the model. Rainfall was not included in the model as a continuous variable, since the values of rainfall were highly right-skewed (over half of the values were zeroes) and there were a few outlier values (S1 Fig). The inclusion of rainfall as a continuous variable in the model would impair the inference for the association between rainfall and dengue incidence. Thus, we classified rainfall into three levels: 0, 0.05-3.85, >3.85 mm. 0.05 mm and 3.85 mm were the minimum and median of the non-zero values of rainfall. We explained the reason in the revision (pages 9-10, lines 177-180).**

**S1 Fig.** Histogram of rainfall.

**R2.4.** Was lag (Intt, 10-21) or lag (Intt, 10-22) included in model 1?

**[Response] We are sorry for the typo. Intt, 10-22 has been changed to Intt, 10-21 in the revision (page 10, line 190).**

**R2.5.** The data on mosquito density was collected once a month from Jan to Mar, but once a week from Jul to Nov. The variable was included into the model using weekly data, how was the monthly data manipulated?

**[Response] We added the following information in the revision：“Since the data on mosquito vector density were collected at the end of month during January and March, the weekly data were available only for the last weeks of these months, while the values of mosquito vector density were missing in other weeks of these months and the missing vales were not imputed.” (page 9, lines 174-177).**

**R2.6.** The results of sensitivity analysis should be submitted in the Sup. Materials.

**[Response] The results of sensitivity analysis have been provided in Supplementary Materials (S5 File, S7 Fig, S3, S4, S5 Tables).**

**R2.7.** The lags of climatic factors were selected based on QAIC. The exposure-response association between climatic factors and dengue incidence should be shown, which is helpful for reader to assess the model, not only using residuals.

**[Response] The values of QAIC of models including natural cubic splines of temperature or relative humidity with different degrees of freedom were provided in S1 Table. We have added the results of the exposure-response associations between climatic factors and dengue incidence in the revision: “It was observed that dengue incidence in general increased with temperature and relative humidity, although the associations were not linear (S5 Fig)” (page 15, lines 285-286).**

**S5 Fig.** Cumulative relative risk of dengue across temperature and relative humidity. (A) Cumulative relative risk of dengue across temperature; (B) Cumulative relative risk of dengue across relative humidity. Medians of temperature and relative humidity were treated as the reference levels for the two variables. Grey regions indicate the corresponding 95% confidence intervals.

**R2.8.** The study used an interrupted time series method to explore the effectiveness of early start of grade III response to dengue. The intervention of response as binary variable, yes or no, was entered into the model. However, I still have concerns on the consistency of the interventions. Responses are separated into six grades, but the core may be the same, what difference may be the intensity. How is the response measured?

**[Response] We apologized that we did not introduce the responses to dengue events of different grades clearly. A reference was cited in the revision to aid understanding of the disparities in the responses of different grades. Admittedly, the results would be more informative if the response was measured in a quantitative way. However, it was extremely difficult to measure the intervention intensity and relevant data were not available. Although the intervention intensity cannot be measured currently and may vary across time, it should be within a certain range for a specific grade of response and distinguished from those for other grades [6]. We mentioned it as a limitation of our study (pages 20-21, lines 398-405).**

**Reference:**

6.	Guangzhou Municipal Health and Family Planning Commission. Preparedness and response plan for dengue fever (2018). 2018; [cited June 25, 2020]. Available from: <http://www.gzcdc.org.cn/uploads/download/20191107/%E5%B9%BF%E5%B7%9E%E5%B8%82%E7%99%BB%E9%9D%A9%E7%83%AD%E7%96%AB%E6%83%85%E5%BA%94%E6%80%A5%E9%A2%84%E6%A1%88%EF%BC%882018%E5%B9%B4%E7%89%88%EF%BC%89.pdf>.

**R2.9:** The aim of the study is to evaluate the effectiveness of early Grade III response to dengue in the high risk area of China, Guangzhou, which is interesting and is benefit for the local health authority and government. The study did a lot of analyses, and the main results were shown. I think other results should also be submitted in Sup. Materials to support the conclusion.

**[Response] We thank reviewer for the favorable comment. As suggested, in addition to S4, S6, S7 Figs and S1, S2, S3, S4, S5 Tables, the results of the associations between climatic variables (i.e. temperature and relative humidity) and dengue incidence have been provided in Sup. Materials in the revision (S5 Fig).**
